# Differential saccade-pursuit coordination under sleep loss and low-dose alcohol

**DOI:** 10.3389/fnins.2022.1067722

**Published:** 2023-02-16

**Authors:** Terence L. Tyson, Erin E. Flynn-Evans, Leland S. Stone

**Affiliations:** ^1^Visuomotor Control Laboratory, Human Systems Integration Division, NASA Ames Research Center, Moffett Field, CA, United States; ^2^Fatigue Countermeasures Laboratory, Human Systems Integration Division, NASA Ames Research Center, Moffett Field, CA, United States

**Keywords:** alcohol, acute sleep deprivation, circadian misalignment, smooth pursuit, saccades, visual motion processing, sensorimotor control, caffeine

## Abstract

**Introduction:**

Ocular tracking of a moving object requires tight coordination between smooth pursuit and saccadic eye movements. Normally, pursuit drives gaze velocity to closely match target velocity, with residual position offsets corrected by catch-up saccades. However, how/if common stressors affect this coordination is largely unknown. This study seeks to elucidate the effects of acute and chronic sleep loss, and low-dose alcohol, on saccade-pursuit coordination, as well as that of caffeine.

**Methods:**

We used an ocular tracking paradigm to assess three metrics of tracking (pursuit gain, saccade rate, saccade amplitude) and to compute “ground lost” (from reductions in steady-state pursuit gain) and “ground recouped” (from increases in steady-state saccade rate and/or amplitude). We emphasize that these are measures of relative changes in positional offsets, and not absolute offset from the fovea.

**Results:**

Under low-dose alcohol and acute sleep loss, ground lost was similarly large. However, under the former, it was nearly completely recouped by saccades, whereas under the latter, compensation was at best partial. Under chronic sleep restriction and acute sleep loss with a caffeine countermeasure, the pursuit deficit was dramatically smaller, yet saccadic behavior remained altered from baseline. In particular, saccadic rate remained significantly elevated, despite the fact that ground lost was minimal.

**Discussion:**

This constellation of findings demonstrates differential impacts on saccade-pursuit coordination with low-dose alcohol impacting only pursuit, likely through extrastriate cortical pathways, while acute sleep loss not only disrupts pursuit but also undermines saccadic compensation, likely through midbrain/brainstem pathways. Furthermore, while chronic sleep loss and caffeine-mitigated acute sleep loss show little residual pursuit deficit, consistent with uncompromised cortical visual processing, they nonetheless show an elevated saccade rate, suggesting residual midbrain and/or brainstem impacts.

## Highlights

- Humans use two systems to follow moving objects with their eyes: pursuit, a smooth continuous eye movement to match target motion, and saccades, quick ballistic jumps to catch up when pursuit lets the eye fall behind the target.
- Pursuit is largely driven by visual motion processing areas in cortex while quasi-reflexive catch-up saccades are largely driven by the superior colliculus and brainstem oculomotor areas.
- These systems are normally tightly coordinated, but little is known about how this coordination may be disrupted by everyday neural stressors.
- Low-dose alcohol (<0.07% BAC) and acute sleep loss (24 h awake) both impair pursuit, but the latter also undermines saccadic compensation, and while caffeine appears to fully mitigate the impacts of sleep loss on pursuit, it may not restore normal saccadic function.
- Low-dose alcohol appears to interfere with cortical visual processing, but one night of sleep loss appears to impact both cortical and sub-cortical visuomotor processing.

## Introduction

Visual tracking of a moving object requires close coordination between smooth pursuit eye movements (pursuit) and catch-up saccades. During steady-state tracking, residual position and velocity errors appear to guide the interplay between smooth and saccadic corrections (Morris and Lisberger, 1987; de Brouwer et al., 2002b; Nachmani et al., 2020). Other factors include prediction, expectation, target size/uncertainty, and perception (Steinbach, 1976; Barnes and Asselman, 1991; Beutter and Stone, 2000; Stone et al., 2000; Krauzlis and Adler, 2001; Stone and Krauzlis, 2003; Hafed and Krauzlis, 2008; Heinen et al., 2016). When the motion is predictable or when the target is cognitively inferred without a visible feature to track, the presence of catch-up saccades is reduced, emphasizing the essential compensatory role of saccades during ocular tracking of the unpredictable visual motion of a moving real-world object.

The precise coordination between the saccadic and smooth pursuit eye-movement systems relies on shared neural processes (Krauzlis and Stone, 1999; Keller and Missal, 2003; Krauzlis et al., 2004; Orban de Xivry and Lefèvre, 2007). Although pursuit can generally only be initiated in response to a moving stimulus (Morris and Lisberger, 1987; see however, Krukowski et al., 2002), positional signals contribute to both steady-state pursuit and catch-up saccades, albeit less so for the former than for the latter. Numerous physiological studies have shown that pursuit (for a review, see Krauzlis, 2004) is primarily driven by motion signals (Lisberger and Westbrook, 1985; Tychsen and Lisberger, 1986) through visual pathways from the Middle Temporal area (MT) (Newsome et al., 1985; Movshon and Newsome, 1996) that culminate in higher-order regions of extrastriate cortex, including the Medial Superior Temporal area (MST) (Dürsteler and Wurtz, 1988; Newsome et al., 1988) and the pursuit region of the Frontal Eye Fields or Frontal Pursuit Area (FPA) (MacAvoy et al., 1991; Tanaka and Lisberger, 2001; Chou and Lisberger, 2004; Leigh and Zee, 2006), before driving pathways through the pons (Suzuki and Keller, 1984; May et al., 1988; Mustari et al., 1988; Glickstein et al., 1994), then to the Ventral Paraflocculus (Miles and Fuller, 1975; Zee et al., 1981; Stone and Lisberger, 1990a,b; Krauzlis and Lisberger, 1991) and Vermis (Suzuki and Keller, 1988a,b; Krauzlis and Miles, 1998; Takagi et al., 2000) of the cerebellum, and ultimately to brainstem output motor pathways via the Vestibular and Fastigial nuclei (Kheradmand and Zee, 2011). Lesions of MT have been shown to disrupt both visual motion perception and initial pursuit acceleration as well as the adjustment of the initial corrective saccade amplitude during pursuit, and exhibits retinotopic spatial tuning (Newsome et al., 1985). Lesions studies in MST and FPA show more pronounced effects, evidenced by sustained uncorrected impairment of steady-state pursuit, and exhibits craniotopic directional tuning (Dürsteler and Wurtz, 1988; Newsome and Paré, 1988; Shi et al., 1998).

When healthy humans track a small target or a larger target with a small central feature, catch-up saccades are used to reduce target/feature position offset from the fovea (Pidcoe and Wetzel, 2006; Heinen et al., 2016, 2018; Shanidze et al., 2016) but also take ongoing target and eye-motion into account (Boman and Hotson, 1992; de Brouwer et al., 2002b), so appear more closely related to the correction of anticipated future offset (Nachmani et al., 2020). Because saccades perturb vision by transiently blurring the retinal image (Castet and Masson, 2000), there is a positional “dead-zone” within which saccades are suppressed to preserve clear vision as long as the target image is close enough to the fovea for high-resolution processing. Mechanistic models (e.g., Ramat et al., 2007; Optican and Pretegiani, 2017; Nachmani et al., 2020; Coutinho et al., 2021) have been proposed to describe this process using the integrated position error up to a threshold prior to triggering catch-up saccades with the threshold circuit controlling when and how often a saccade is made (saccade rate) and separate downstream circuits controlling the size of the correction (saccadic amplitude). Physiological studies suggest that the rostral pole of the Superior Colliculus (rSC) plays a critical role in the triggering of both small saccades and small smooth corrective accelerations (Basso et al., 2000; Krauzlis et al., 2000; Hafed et al., 2009; Hafed, 2011). The SC projects both to omnipause neurons in the Raphe Interpositus Nucleus and to burst neurons in the pontine and medullary reticular formation (Sparks et al., 2002; Ramat et al., 2007; Takahashi et al., 2022) with tonic activity within rSC involved in suppressing saccades to promote fixation (Munoz and Wurtz, 1993a,b; Wurtz and Optican, 1994) and to maintain a stable “dead-zone” with smooth corrections as needed during steady-state tracking (Basso et al., 2000; Krauzlis et al., 2000) and phasic activity involved in generating small saccades (Hafed et al., 2009; Hafed, 2011). The exact interplay between these two competing roles is not yet fully understood. The well-organized spatial map of saccade sizes within the rSC is converted into the temporal code within brainstem circuits (for a review, see Sparks et al., 2002) that control saccadic amplitude likely by having the locations coding larger saccades exerting larger synaptic strength in their inputs to burst neurons (Moschovakis et al., 1998). The output of burst neurons that drives the pulse signal drive to motoneurons is then integrated to generate the step signal to motoneurons to hold eccentric gaze as well as the internal feedback of the ongoing saccadic displacement to control the endpoint and thus the amplitude of the saccade (Robinson, 1975; Sparks et al., 2002; Ramat et al., 2007; Optican and Pretegiani, 2017).

### Effect of sleep loss

The coordination between pursuit and saccades requires healthy neural processing and thus may be vulnerable to various conditions of mild neural impairment, including sleep loss. Previous studies of acute sleep loss ranging from 16 to 36 h have shown decrements in pursuit as well as an increase in the frequency of catch-up saccades during steady-state tracking (Fransson et al., 2008; Stone et al., 2019). Furthermore, the saccadic peak velocity of horizontal pro-, anti-, and memory-guided saccades is decreased after a single night of sleep deprivation (Zils et al., 2005). Moreover, circadian rhythms appear to modulate saccadic performance, with longer latencies and slower peak velocities during the circadian trough (Stone et al., 2019). Chronic sleep restriction has also been shown to impact pursuit and saccades (Evans et al., 2021). In sum, sleep loss and circadian misalignment adversely impact the performance of both pursuit and saccades, but the existing literature has not addressed their coordination (or lack thereof) during steady-state tracking.

### Effect of alcohol

It is well known that alcohol can cause severe sensorimotor disruption (gaze-paretic nystagmus, ataxia, slurred speech) at Blood-Alcohol Concentrastions (BACs) above the standard legal limit of 0.08%, forming the rationale for law enforcement’s field-sobriety tests. A number of scientific studies have documented pursuit deficits at BAC above 0.04% during the tracking of sinusoidal or step-ramp motion (Fransson et al., 2010; Roche and King, 2010) and, more recently, significant impairment has even been demonstrated at BACs as low as the 0.005–0.015% range (Tyson et al., 2021). Additionally, saccade dynamics are impacted by low-dose alcohol; specifically, a reduction in peak saccadic velocity was shown at BAC levels between 0.025 and 0.12% (Lehtinen et al., 1979; Vorstius et al., 2008; Fransson et al., 2010; Roche and King, 2010; Tyson et al., 2021) coupled with an increase in saccadic latency (Vorstius et al., 2008; Fransson et al., 2010; Roche and King, 2010). In sum, low-dose alcohol adversely impacts the performance of both pursuit and saccades, but the existing literature has not addressed their coordination (or lack thereof) during steady-state tracking.

The current study seeks to elucidate how the coordination of pursuit and saccades during steady-state tracking may differ under various conditions of mild neural impairment, more specifically during acute sleep loss (ASL), acute sleep loss with a caffeine countermeasure (ASL+C), chronic sleep restriction (CSR), and low-dose ethanol (EtOH), with the aim of determining if they produce disparate patterns of behavioral effects that would indicate differential impacts of these stressors on the neural substrates described above.

## Materials and methods

### Participants

In the current study, we re-analyzed data from four separate previous experiments, each examining distinct treatment effects on sensorimotor performance during the same human ocular tracking task. The primary treatment effects were acute sleep loss (ASL), acute sleep loss with caffeine administration (ASL+C), chronic sleep restriction (CSR), and acute low-dose alcohol administration (EtOH). For information on participant demographics, see Table 1. Seven of the 12 participants from the ASL experiment returned the following calendar year for the ASL+C experiment and were used for the within subject analyses. However, two additional participants were recruited for the caffeine study, hence a total of nine ASL+C participants were used for the across-subjects analyses. The EtOH experiment was performed using an independent cohort with the exception of one participant who participated in all of the studies except for CSR. The single participant who participated in three of the four studies was evaluated as a case study to illustrate the differential mechanisms of saccadic compensatory behavior across the various treatment conditions within the same subject. The CSR study was performed using an independent cohort from the other studies.

**TABLE 1:** Participant demographics and treatment conditions (mean ± SD).

	Acute sleep loss (ASL)	ASL with caffeine (ASL+C)	Chronic sleep restriction (CSR)	Low-dose alcohol (EtOH)	
				Female	Male
*N*	12 (6 females)	9 (4 females)	12 (6 females)	8	8
Age (yrs old)	24.8 ± 5.6	23.8 ± 3.4	23.0 ± 3.9	25.1 ± 2.0	26.0 ± 4.1
Weight (kg)	67.8 ± 13.5	71.4 ± 14.8	63.2 ± 13.1	63.6 ± 6.6	74.2 ± 8.2
Height (cm)	170.1 ± 10.8	172.3 ± 12.9	166.2 ± 10.9	162.9 ± 8.4	176.5 ± 5.6
BMI (kg/m^2^)	23.3 ± 3.1	23.9 ± 3.3	22.7 ± 2.8	24.0 ± 2.0	23.8 ± 2.3
Sleep duration (h)	7 h 48 ± 16 min	7 h 56 ± 22.7 min	7 h 24 ± 26 min	8 h 33 ± 36 min	8 h 22 ± 23 min
Treatment dose	∼24 h awake	170.7 ± 38.2 mg*	4 h 25.5 ± 19.9 min^§^	2.16 ± 0.23^†^	2.97 ± 0.33^†^

yrs, years; kg, kilograms; cm, centimeters; BMI, body mass index; mg, milligrams.

^†^Standard drinks of alcohol for the 0.06% target peak BAC condition.

*Total caffeine dose overnight taken hourly at 0.3 mg/kg.

^§^Sleep duration under the 5-h sleep restriction condition.

Note that the SDs reported for ASL are sample SDs, whereas the SDs reported in a previous publication are population SDs (Stone et al., 2019).

The three sleep studies took place in the Fatigue Countermeasures Laboratory and the EtOH study took place in the Visuomotor Control Laboratory at the National Aeronautics and Space Administration (NASA) Ames Research Center and were approved by the center’s Human Research Institutional Review Board (HRIRB) under protocols HRI-325, HRI-17-09, HRI-336, and HRI-349. All participants provided written informed consent and we have followed the standards set by the Declaration of Helsinki.

### Exclusionary criteria

We used the same exclusionary criteria for both the ASL and ASL+C experiments (under section “methods” see section “Selection/exclusion criteria,” Stone et al., 2019). In the EtOH experiment, the exclusionary criteria differed in certain aspects from the two ASL experiments (Tyson et al., 2021).

For the CSR experiment, participants were required to abstain from nicotine, marijuana, and drugs of abuse throughout the study. Participants were limited to one caffeinated beverage and one alcoholic beverage during the washout weeks. During the experimental weeks, participants were instructed to completely abstain from alcohol and caffeine. To ascertain compliance, a urine toxicology test was administered upon arrival to the laboratory. Participants were excluded if they tested positive for caffeine, nicotine, marijuana, amphetamines, barbiturates, benzodiazepines, cocaine, MDMA, methadone, methamphetamines, opiates, or oxycodone. Additionally, participants were excluded if they had a body mass index (BMI) of greater than 30, had suffered a head injury that resulted in a concussion or a loss of consciousness, had travelled outside of the Pacific Standard Time (PST) zone within the past 3 months, had regularly worked night shifts in the past 2 years, had a history of psychiatric or psychological disorders, or had ever consumed the anti-acne medications Oral Retin-A, Accutane, or Tetracycline. Participants were excluded if they scored > 5 on the Pittsburgh Sleep Quality Index (PSQI; Buysse et al., 1989), if they scored < 31 or > 69 on the Morningness-Eveningness Questionnaire (MEQ; Horne and Ostberg, 1976), if they scored > 40 on the State Trait Anxiety Inventory (STAI; Spielberger et al., 1983), > 10 on the Beck Depression Inventory-1A (BDI-1A; Beck and Steer, 1993), or if they scored above any of the following primary symptom dimensions of the Symptoms Checklist 90-R (SCL-90-R; Derogatis, 1994): > 1.25 on Anxiety, Depression, or Paranoid Ideation, > 1 on Hostility or Psychoticism, or > 0.75 on Phobic Anxiety. Similarly, participants were excluded if they scored > 70 on Depression or > 75 on Hypomania, Psychopathic Deviance, or Schizophrenia on the Minnesota Multiple Phasic Personality Inventory-2 (MMPI-2; Butcher et al., 1989).

### Oculomotor task

Under all experimental conditions described in subsequent text, we used an efficient and randomized oculomotor behavioral task based on the classic Rashbass step-ramp paradigm (Rashbass, 1961) adapted to accommodate a full sampling of polar angles (Krukowski et al., 2003; Krukowski and Stone, 2005) using 90 or 180 trials per run corresponding to a trial every 2 or 4 degrees around the clock. Each trial started with a fixation target in the middle of the screen. The participant was instructed to initiate the trial by a manual button press on a game controller when they were ready after fixating the central target. After a random amount of time between 200 and 5,000 ms (truncated exponential distribution), the 0.2-deg target jumped 3.2–4.8 deg away from the fixation point, immediately moved back at a constant speed (16, 18, 20, 22 or 24 deg/s) toward the fovea, and then onward for a random amount of time from 700 to 1,000 ms before disappearing. Participants were instructed to keep their eyes on the target in the center without blinking and then to follow it as best they could once it started moving until it disappeared. The target would then reappear in the central location, awaiting the initiation of the next trial by the participant. We used a video-based, table-mounted, pupil-tracking system with an accuracy of ∼0.5 deg and a precision of ∼0.2 deg, and an HD-resolution 144 Hz BenQ™ model XL2420Z display (Liston et al., 2016). We calibrated the eye tracker by having the subjects fixate nine locations on a 3 × 3 Cartesian grid to derive a six-parameter affine transformation from camera to world coordinates (Beutter and Stone, 1998). Using a chin and forehead rest to minimize head movements, seated participants viewed the target binocularly from 46 cm and performed repeated runs at different values of time awake in studies 1ab and 2, and at different blood-alcohol levels in study 3.

### Oculometric analysis

We used established analyses to measure a large set of oculomotor metrics (described in detail in Stone et al., 2019) but here we focused on only three (pursuit gain, saccade amplitude, and saccade rate during the steady state interval 400–700 ms after motion onset) to compute the derived measures of ground gained and ground lost (see below). Prior to analysis, we computed eye-velocity traces (low-pass filtered with a simple 3-point non-causal digital filter with coefficients ¼, ½, ¼), then detected and removed saccades using a template-correlation method described in detail elsewhere (Liston et al., 2013), modified to apply a bi-phasic saccade template tailored for the higher spatio-temporal fidelity of our 250-Hz eye tracker. We were thus able to reliably detect and remove saccades down to approximately one-eighth of a degree in amplitude (limited by tracker noise). We then computed the following oculometric measures using MATLAB™ (R2017a, The MathWorks, Natick, MA, USA):

• Steady-state gain was defined as the median across trials of the mean eye speed of the saccade-free component of the steady-state tracking response, projected along the target direction and divided by the target speed. Trials for which steady-state velocity was negative, or for which there was less than 80 ms of saccade-free pursuit in the steady-state interval, or for which there was a blink during the steady-state interval, or for which the steady-state eye speed was unstable (SD > 8 deg/s) were excluded from the computation (∼10% of trials).
• Saccadic rate was defined as the total number of catch-up saccades occurring in the steady-state tracking interval divided by the total steady-state tracking time (300 ms per trial plus any added lead time if a saccade onset preceded and the saccade spanned the initial interval boundary). Trials with blinks in the steady state were excluded, but this occurred rarely.
• Saccadic amplitude was defined as the median amplitude of the forward saccades occurring in the steady-state tracking interval.

### Experiment 1a: Acute sleep loss (ASL)

The study methodology was outlined in extensive detail in a previous manuscript (Stone et al., 2019) and is summarized below. The effects due to time awake (i.e., homeostatic sleep pressure) and circadian rhythm cannot be definitively distinguished in this study.

Subjects were instructed to complete a regular 14-day sleep-wake schedule prior to lab testing. During this period, they were instructed to maintain an individually designed sleep-wake schedule, with 8.5 h of time in bed. To verify compliance, they wore an actigraph (Actiwatch Spectrum, Respironics Inc., Bend, OR, USA) on their non-dominant wrist to quantify the duration and quality of sleep as well as the degree of light exposure throughout the day. Additionally, timestamped voicemails were recorded both prior to their sleep and immediately after awakening, as well as having each event recorded in a sleep diary.

After completing the 14-day sleep-wake schedule, subjects would visit the NASA Ames Research Center to complete an overnight lab study. During this visit, we conducted a constant-routine protocol (CR)(Duffy and Dijk, 2002) to lessen the influence of external confounds in order to more effectively measure changes in oculomotor behavior due specifically to sleep deprivation and circadian rhythms.

Subjects completed 2–5 daytime baseline runs and 8 nighttime runs of the ocular tracking task, with the nighttime measurements beginning near the subjects’ habitual bedtime.

### Experiment 1b: Acute sleep loss with acute low-dose caffeine administration (ASL+C)

In addition to the above procedures, in this study, during the nighttime, subjects ingested a caffeine pill hourly (average caffeine dose per pill: 21.3 ± 4.8 mg or 0.3 mg/kg for each participant), totaling 8 caffeine pills. We used small hourly doses of caffeine as it was previously shown to be sufficient to sustain alertness and performance during circadian misalignment (Wyatt et al., 2004). Caffeine pills were created by a licensed pharmacist using pure caffeine from a compounding pharmacy. Pills were administered in conjunction with the hourly food ration.

### Experiment 2: Chronic sleep restriction (CSR)

For the at-home portion of the study, subjects completed a washout condition on their first week, following a treatment condition on their second week. The washout condition consisted of a habitual sleep satiation regime where subjects were instructed to achieve 9-h of sleep each night. In the subsequent treatment condition week, subjects were randomly assigned to either the 9-h or 5-h sleep condition, the order in which was counterbalanced across subjects. Following the at-home preparation period, subjects completed a one-day lab study in which repeated measurements of ocular tracking performance were collected. After the completion of testing for the first treatment condition, subjects completed another week of at-home preparation and subsequent lab days for the other treatment condition. Subjects abstained from caffeine and alcohol throughout the study.

### Experiment 3: Acute low-dose alcohol administration (EtOH)

This study methodology was described in detail in a previous manuscript (Tyson et al., 2021). Briefly, participants completed a three-day at-home phase followed by a two-day lab phase. During the at-home portion, subjects maintained a sleep schedule (∼8.5 h of sleep per night at the same time of day) and abstained from caffeine and alcohol. Following the at-home phase, subjects were brought to the lab to complete repeated measures of ocular tracking performance before and after alcohol administration. The alcohol dosage was determined by weight and sex with target peak blood alcohol concentrations of either 0.02 or 0.06%. Subjects were randomly assigned to receive the lower or higher initial dose on the first or second lab day. During each lab day, subjects completed 3 pre-dose runs and 5–13 post-dose runs of the ocular tracking task.

### Saccadic compensation metrics

**FIGURE 1:**
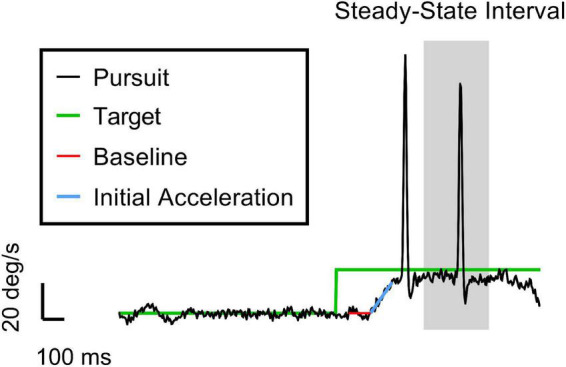
An example trial of the ocular tracking response in our task. The vertical and horizontal axes are velocity and time, respectively. The black and green traces are the eye and target velocities (20 deg/s target speed for this trial), respectively. The red and blue lines represent the best hinge fit, where the breakpoint indicates the pursuit latency when the eye starts to accelerate. The slope of the blue line gives the initial acceleration of the eye. The grey region is the 300-ms interval of time in which we calculate the closed-loop pursuit gain and the saccade metrics (i.e., rate and amplitude). We chose 400–700 ms as the steady-state (SS) interval to isolate these catch-up saccades from both tracking initiation and any potential behavioral disengagement due to anticipation of the end of the trial. Note the initial clean smooth initiation typical of the Rashbass design. The first saccade in the trace was a late initial corrective saccade related primarily to the imperfect smooth initiation. The second saccade is deemed a true steady-state catch-up saccade since it occurred during the steady-state interval, suggesting that the saccade was closely related to the sustained suboptimal closed-loop steady-state pursuit gain (<1).

We used a subset of oculometrics (Liston and Stone, 2014; Stone et al., 2019) – specifically, pursuit gain, saccadic rate, and saccadic amplitude – to compute the cumulative lost ground (in degrees of visual angle) due to a pursuit deficit during the closed-loop steady-state tracking interval (400–700 ms after target motion onset) and any associated ground recouped by catch-up saccades during the same time interval. See Figure 1 for a trial breakdown.

To compute the average ground lost across a run, we used the following formula:


GroundLost=6deg⋅(1-G)

*G* is the median pursuit gain across trials during steady-state tracking. 6 degrees represents the average distance traversed by the target in the steady-state analysis window across a given 90–180 trial block with 20 deg/s being the average of target speed and 0.3 s the duration of the steady-state tracking interval.

To compute the average ground recouped across a run, we used the following formula:


GroundRecouped=fA⋅(0.3s)

with *f* and *A*, the median rate (in Hz) and amplitude (in deg) of catch-up saccades during steady-state tracking. Because saccade rate was highly quantized and variable on a trial-by-trial basis, we performed the above analyses averaged across all trials within a run to generate more reliable data and then performed statistical tests across our cohort of participants.

### Statistical analysis

All data analysis routines were performed using MATLAB (versions R2017a or R2020a, MathWorks, Natick, MA, USA), GraphPad Prism (version 9, GraphPad Software, San Diego, CA, USA), and Excel (Microsoft Corp., Redmond, WA, USA). We computed dose-responses within-subject as the linear regression slopes of ground lost and recouped as a function of time awake (TA) or blood-alcohol concentration (BAC). We then compared across-subject, for each experiment, the mean slopes between ground lost and ground recouped using a paired *t*-test (two-tailed). We also evaluated independently if the ground lost and ground recouped were significantly greater than zero (i.e., null dose-response) using a one-sample *t*-test (one-tailed) given our *a priori* assumption of some pursuit impairment and some saccadic compensation. To compute the effects on our constituent metrics, we performed one-sample *t*-tests for each metric and each specific stressor. In addition, we performed Independent-measures *t*-tests to reveal any differences in the effects between stressors.

For the circadian analysis, performance data were plotted as a function of circadian phase (i.e., phase-locked with peak melatonin concentration determined from hourly saliva samples per Stone et al., 2019) and fit with a two-parameter cosine model (phase and amplitude) using the least-squares method. The objective function was defined as the residual sum-of-squares between the cosine model and the performance data; this objective function was then minimized using a nonlinear programming solver (MATLAB’s fminsearch). The across-subject mean phase and amplitude estimated from the minimization routine were statistically compared between the ground lost and ground recouped data using a paired *t*-test (two tailed).

### Circadian simulation

**FIGURE 2:**
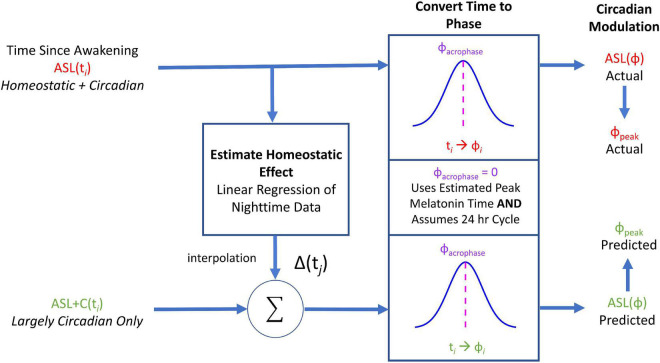
A flowchart of the circadian phase computations for actual ASL and predicted ASL using ground recouped as the performance measure of interest. Predicted ASL was computed by adding the homeostatic estimate from the ASL data to the circadian estimate from ASL+C. This simulation analysis served as an internal self-consistency validation of our ASL phase estimates. The observed phase alignment from this analysis suggests that the circadian phase differences between ASL and ASL+C could largely be attributed to homeostatic sleep pressure.

To recreate the circadian phase variance observed in ground recouped under ASL, we used the time awake data sets of the ASL and ASL+C conditions to estimate the homeostatic and circadian components, respectively, and recombined these two estimates to simulate the ASL modulation response. Figure 2 provides an overview of the workflow of our simulations of the circadian modulation phase observed during ASL. First, we performed linear regression of the nighttime ground recouped ASL data to estimate the rate of the homeostatic performance decrement due to increasing sleepiness during forced wakefulness. We then interpolated to enable the addition of the appropriate decrement to the time awake data of the ASL+C data set. This reconstituted time-awake data set was then converted to circadian phase using melatonin acrophase as the reference marker (Stone et al., 2019). After conversion, we then fit the ground recouped of this new data set with a best-fitting cosine using least-squares and estimated the phase.

To evaluate the degree in which we properly reconstructed the phase variance in ASL, we compared the estimated phase of this prediction with the observed phase of ASL data using a paired two-tailed *t*-test across subjects. A significant difference would indicate that the prediction failed.

## Results

### Case study

**FIGURE 3:**
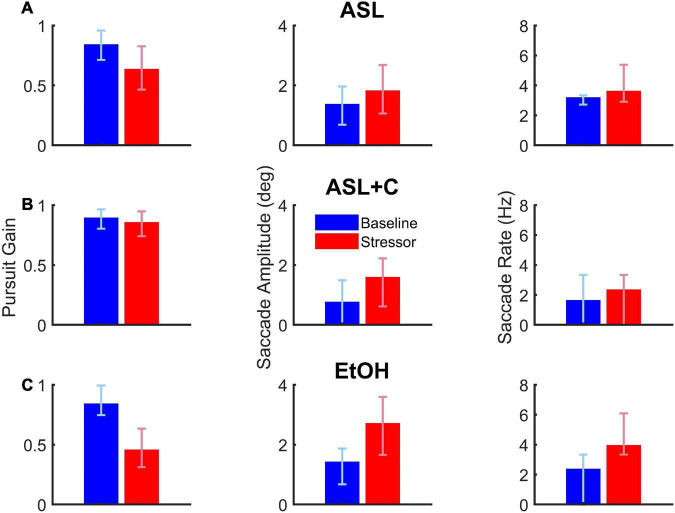
The effects of acute sleep loss without (**A**, ASL, 24.5 h awake) and with a caffeine countermeasure (**B**, ASL+C, 22.6 h awake) and of low-dose alcohol (**C**, EtOH, 0.033% BAC) for a single example participant. Each row shows summary data of the three raw oculometrics (i.e., pursuit gain, saccadic amplitude, and saccadic rate) used to derive the saccadic compensation metrics (i.e., ground lost and group recouped) for each of the above three conditions. The columns from left to right show bar charts of pursuit gain and saccadic amplitude with the red and blue vertical bars indicating the median of the stressor and baseline distributions of a 90–180 trial run, respectively, and the error bars indicating the interquartile range. For saccade rate, the vertical bars show the total number of saccades divided by the total duration of the steady-state period (i.e., 300 ms per trial) across all 90–180 trials, with the error bars indicating the interquartile range for the trial-by-trial saccade rate across trials. Pursuit gain is impaired in both the ASL and EtOH conditions. Saccadic rate and amplitude, however, show larger increases in the EtOH condition, suggesting more effective compensatory neural mechanisms under low-dose alcohol as compared with a night of sleep loss. Caffeine appears to remedy most of the pursuit deficit, but saccadic behavior nonetheless appears abnormally elevated with respect to baseline.

**FIGURE 4:**
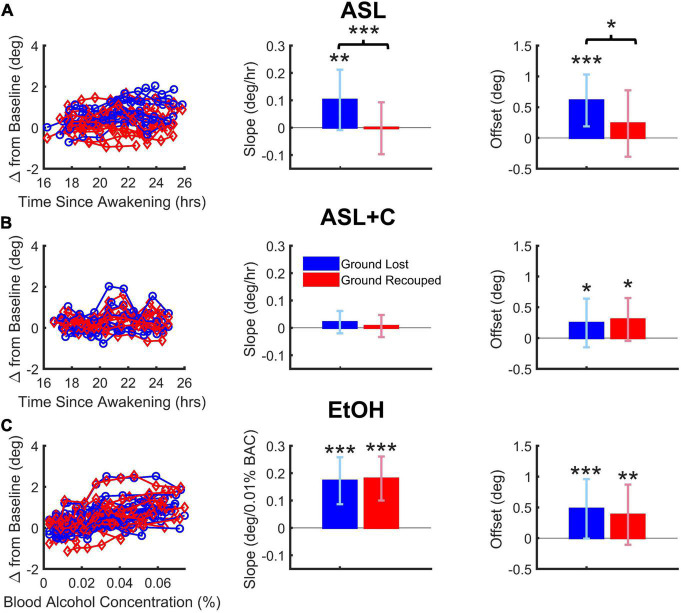
Dose-dependent and overall effects on saccade-pursuit coordination. Vertical and error bars represent the across-subject mean and standard deviation (SD). **(A)** Under ASL, there was a significant difference between ground lost and recouped, reflected in both the dose-response sensitivity to time awake (middle column) as well as the overall effect across the range of time-awake doses tested (righthand column), indicating at best only partial saccadic compensation. **(B)** Under ASL+C, both the ground lost and gained became insensitive to increased wakefulness (middle) but there is a hint of a small non-dose-dependent overall saccadic overcompensation (right). **(C)** Under EtOH, there was a large dose-dependent increase in ground lost with increasing BAC dose, however, the ground recouped showed a dose-dependent increase sufficient to fully counteract that loss (middle) with no overall difference as well (right). *p < 0.05, **p < 0.01, and ***p < 0.001.

This case study illustrates the differential outcomes of saccade-pursuit coordination between three experimental conditions (ASL, ASL+C, and EtOH) for the single subject who participated in these three experiments (Figure 3). Under EtOH (bottom row), this participant showed a serious decrease in pursuit gain (–46.0%) with large compensatory increases in both saccade rate (68.2%) and amplitude (91.9%). Under ASL (top row), this participant again exhibited a serious pursuit gain reduction (–24.5%) but more moderate increases in both saccadic rate (13.7%) and amplitude (33.6%), even when scaled for the difference in the pursuit deficit, suggesting that ASL also compromised saccadic compensation. For ASL+C (middle row), the pursuit gain recovered most of its baseline vigor (only –4.2% compared to baseline); yet increases in both the rate and amplitude of the catch-up saccades persisted, even more pronounced (44.1 and 112.7%, respectively) than with ASL alone, indicating a maladaptive saccadic response and suggesting that caffeine may be driving saccades to overcorrect. This basic pattern was confirmed across our sample of participants (see Figure 4).

### Dose-dependent saccadic compensation

Figures 4A–C shows the dose-dependent effects on the total accumulated position error (ground lost in blue) and the total position correction effected by catch-up saccades (ground recouped in red) during steady-state pursuit for ASL, ASL+C, and EtOH conditions, respectively, with the raw data from all participants on the left, the mean slope of their dose-response trends in the middle, and the overall mean responses across the range of doses tested on the right.

For EtOH, there was a highly significant pursuit deficit as ground lost showed a significant dose-dependent effect quantified as the mean slope of the linear trend (*t*(15) = 8.04, *p* < 0.0001). However, there was also highly significant dose-dependent compensation from catch-up saccades (*t*(15) = 8.96, *p* < 0.0001) such that there was no significant difference between the dose-response slopes for ground lost and ground recouped (*t*(15) = –0.33, *p* = 0.74) or overall effects averaged across subjects (*t*(15) = 0.75, *p* = 0.47). This shows that, while alcohol at doses below a BAC of 0.07% significantly impairs the pursuit system in a dose-dependent manner, it does not impair saccadic compensation.

For ASL, there was a significant pursuit deficit as ground lost showed a significant mean dose-dependent slope (*t*(11) = 3.19, *p* = 0.004). Furthermore, the slope for ground lost was significantly larger than that for ground recouped (*t*(11) = 5.75, *p* < 0.0002), which itself did not reach significance (*t*(11) = –0.07, *p* = 0.47), resulting in significantly smaller overall ground recouped than ground lost across wakeful nighttime hours (*t*(11) = 2.66, *p* = 0.022). This shows that staying up all night significantly impairs not only the pursuit system, but also the saccadic system’s ability to compensate.

**FIGURE 5:**
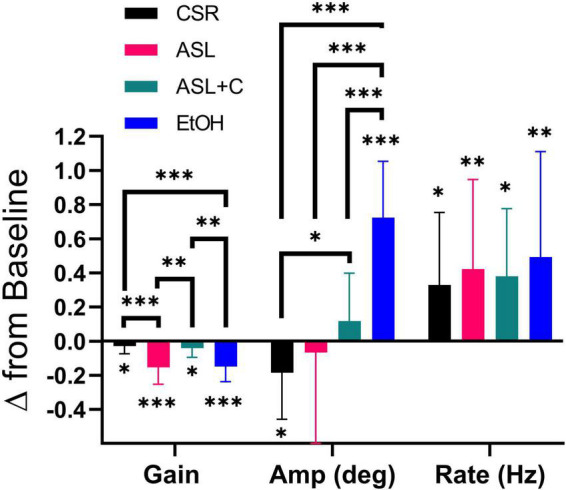
Differential pursuit and saccade performance with sleep loss and low-dose alcohol. The bar chart depicts the average and standard deviation (SD), with color indicating the experimental condition. Significant deficits in pursuit gain were revealed across all conditions. EtOH largely recouped the loss with an increase in saccade rate and amplitude, whereas ASL showed an increase in saccade rate and a highly variable (insignificant) amplitude change, yielding weaker compensation. CSR showed a smaller pursuit gain deficit, coupled with an unnecessarily large increase in saccade rate that was tempered by a decrease in saccade amplitude. With ASL+C, pursuit gain and saccadic amplitude were similar to baseline, but saccadic rate nonetheless increased unnecessarily. *p < 0.05, ^**^p < 0.01, and ^***^p < 0.001.

For ASL+C, slope estimates of the ground lost (*t*(8) = 1.52, *p* = 0.083) and ground recouped (*t*(8) = 0.49, *p* = 0.32) were not significantly higher than zero, and there was no significant difference between the two (*t*(8) = 1.24, *p* = 0.25). There was also no significant difference between the overall mean ground lost and recouped across wakeful nighttime hours (*t*(8) = –0.50, *p* = 0.63). This shows that, while staying up all night significantly impairs the pursuit system, caffeine can effectively fully counteract this sleep and circadian effect, although the saccadic system’s status remains unclear in this analysis as there is almost no pursuit loss to compensate for (see however, Figure 5 below). However, the fact that overall ground recouped is significantly higher than baseline (*t*(8) = 2.60, *p* = 0.0159) and even, on average, 23.0% (albeit insignificantly) larger than the ground lost (an overcompensation not observed in the other conditions) suggests that there may also be a small, uncompensatory saccadic boosting response to caffeine, that is unrelated to sleep loss or circadian misalignment, consistent with the observed significant increase in saccadic rate, despite the minimal pursuit impairment under ASL+C (see Figures 3, 5).

### Comparison of saccadic compensation across three stressors

We also examined pursuit and saccadic tracking responses during chronic sleep restriction (CSR) albeit for a single dose (5 h of sleep per night). Below we compare the effects of CSR at that dose with the ASL response near the nadir of circadian phase (23 h awake) and with the EtOH response at a BAC of 0.065% (Figure 5). While all three conditions caused significant impairment of pursuit (decreased gain), the CSR effect was much more muted than those for ASL and EtOH (ASL: *t*(11) = –5.3195, *p* < 0.0002; CSR: *t*(11) = –2.3187, *p* = 0.0204; EtOH: *t*(15) = –6.8642, *p* < 0.0001). All three conditions generated significant increases in saccadic rate (ASL: *t*(11) = 2.7933, *p* = 0.0088; CSR: *t*(11) = 2.6757, *p* = 0.0108; EtOH: *t*(15) = 3.1950, *p* = 0.003). However, given the small magnitude of the pursuit impairment under CSR relative to baseline performance, the large increase observed in saccadic rate was unnecessary. Furthermore, unlike EtOH and ASL which show a significant increase (*t*(15) = 8.7705, *p* < 0.0001) or no-change in saccadic amplitude (*t*(11) = –0.4118, *p* = 0.3442), respectively, CSR shows a significant *decrease* in saccadic amplitude (*t*(11) = –2.355, *p* = 0.0191). This surprising finding may nonetheless just be compensatory for the unnecessarily large increase in saccadic rate. With caffeine, saccadic amplitude does not change significantly (*t*(8) = 1.2532, *p* = 0.1228) yet still pursuit gain decreases and saccadic rate increases significantly (Pursuit gain: *t*(8) = –2.2845, *p* = 0.0259; Saccadic rate: *t*(8) = 2.8936, *p* = 0.0101). Hence, the differential response to the four conditions is most clearly illustrated by the qualitative differences in the observed effects on the amplitude of catch-up saccades (see middle quadruplet in Figure 5).

### Circadian-dependence of saccade-pursuit coordination

**FIGURE 6:**
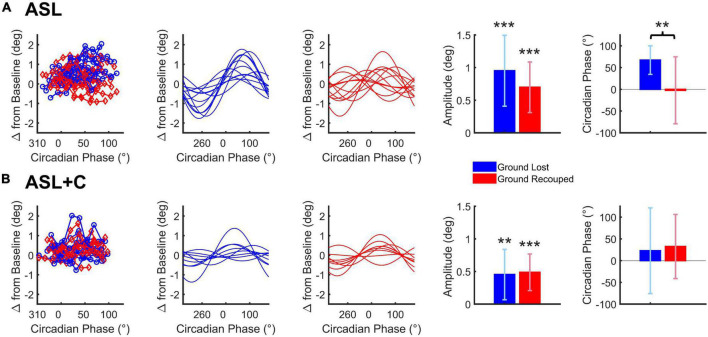
Cosine analysis of ground lost vs. ground recouped (depicted in blue and red, respectively) for ASL **(A)** and ASL+C **(B)**. Raw data for ground lost and ground recouped data are shown in the leftmost panels with cosine fits of the ground lost and ground recouped in separate adjacent panels. Bar charts represent the across-subject average (±SD) of the best-fitting amplitude and phase parameters. *p < 0.05, **p < 0.01, and ***p < 0.001.

Figure 6 shows our circadian analysis for ground lost and ground recouped under ASL and ASL+C. The analysis revealed distinct profiles in modulation and phase of the compensatory behavior and saccade-pursuit coordination between the two conditions.

For ASL, there was a significant circadian modulation in the ground lost and ground recouped over the 24-h cycle (*t*(11) = 6.0668, *p* < 0.0001 and *t*(11) = 6.2290, *p* < 0.0001, respectively) but the amplitude of the ground recouped appeared smaller than that for ground lost, although the difference was not quite significant (*t*(11) = 2.1395, *p* = 0.0557) likely due to the large inter-subject variability. More importantly, while the circadian phase for ground lost was well behaved peaking around +60° for most participants, that for ground recouped was more variable and, on average, closer to 0°. This phase misalignment was significant (*t*(11) = 3.4216, *p* = 0.0057) so the saccadic system is not providing fully effective compensation for the circadian modulation under ASL.

For ASL+C, the circadian modulation in ground lost remained significant albeit smaller than without caffeine (*t*(8) = 3.5245, *p* = 0.0039). The modulation in ground recouped was also significant (*t*(8) = 5.2185, *p* = 0.0004). These two amplitudes were not significantly different (*t*(8) = −0.3113, *p* = 0.7635), and their phases were well aligned (*t*(8) = –0.4997, *p* = 0.6308). Thus, in the presence of caffeine, the saccadic system fully compensates for the significant circadian modulation in pursuit performance, although the (insignificantly) larger mean modulation in ground recouped suggests a hint of overcompensation.

### Circadian phase variability in saccadic compensation

**FIGURE 7:**
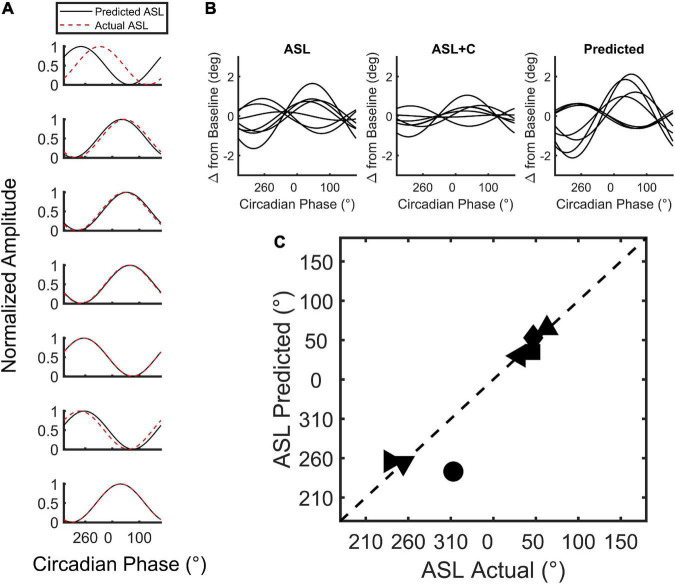
Simulation of circadian phase variability in ground recouped during ASL. **(A)** Plot of the min-max normalized cosine fits of the predicted (red) and actual (black) ASL modulation for all seven subjects tested. **(B)** The unnormalized best-fit modulation of the seven subjects for the actual and predicted ASL. Note that the modulation phase of the prediction qualitatively resembles the actual modulation of the ASL data with one exception. **(C)** Two-dimensional scatterplot of predicted versus actual circadian phase with each point depicting a subject.

We used a model (Figure 2) to simulate the phase of the circadian response during ASL. The model combines an estimate of circadian cycling (amplitude and phase) based on the modulation during ASL+C with an estimate of the homeostatic sleep drive based on the nighttime linear trend during ASL to simulate the phase variability observed in ASL (Figure 7). The simple addition of these two signals allowed us to predict the phase shift observed in ASL. The cosine predictions were highly correlated with the actual fits of the ASL data for six of the seven subjects (*r*^2^ ≥ 0.91 see Figure 7A) with the correlation for the remaining subject (top trace) with the outlier phase prediction being lower (*r*^2^ = 0.12). There was no significant difference between the predicted and actual phase for both the entire within-subject cohort (*t*(6) = 0.7405, *p* = 0.4869), or when excluding the outlier (*t*(5) = –0.6256, *p* = 0.559). Thus, the simulated predictions were able to recreate the phase variability observed in the apparent circadian variability of ASL from the circadian modulation during ASL+C and the estimated homeostatic effect from ASL nighttime time-awake linear trends.

## Discussion

We found that the coordination of the pursuit and saccadic systems is differentially affected by three different stressors and one pharmacological countermeasure. Low-dose alcohol (our EtOH condition) generates a large reduction in pursuit gain, but the resulting accumulated position error over time is nearly fully corrected by catch-up saccades such that the fovea catches up and, on average, is effectively pointed at the target during steady-state tracking. Acute sleep loss and circadian misalignment (our ASL condition) also generates a large reduction in pursuit gain. However, the accumulated position error over time is not fully recouped due to inadequately sized catch-up saccades, such that the fovea is systematically pointing slightly behind the target during steady-state tracking. Caffeine (our ASL+C) appears to eliminate most of the pursuit deficit related to time awake, although saccadic rate remains unnecessarily elevated, perhaps via a direct effect of caffeine on the saccadic system unrelated to its role as a countermeasure for sleep loss (Connell et al., 2017) or perhaps the ASL effect on saccadic rate is not mediated by adenosine pathways (Blanco-Centurion et al., 2006). Chronic sleep restriction (our CSR condition) shows a much milder reduction in pursuit gain, suggesting an adaptive mechanism that at least partially compensates for sleep deprivation when it becomes chronic. As with ASL+C, there is an unnecessary increase in saccade rate that may trigger the observed apparently compensatory *decrease* in saccade size, emphasizing that the adverse impacts of sleep restriction on sensorimotor control are not fully overcome by chronic adaptation, just as caffeine does not suppress the effects of acute sleep loss on saccade rate.

The disparate nature of our four behavioral test conditions highlights the fact that an examination of a wide array of largely independent parameters of oculomotor responses (multidimensional oculometrics) not only allows for the sensitive detection of mild, subclinical performance deficits, but also supports the making of specific distinctions about the potential disparate causes of these deficits (Liston et al., 2017; Tyson et al., 2019). It also suggests that the observed differential patterns of behavioral effects reflect perturbed neural processing within different neural loci (Leigh and Zee, 2006; Ramat et al., 2007; Stone et al., 2019). Lastly, it must be pointed out that even when saccades compensate fully for the position error that results from poor pursuit gain, saccades cannot reduce the retinal slip (motion blur) that occurs with the resulting saccade-mediated foveation, thus saccadic compensation is always only a partial remedy for the loss of pursuit and associated loss of dynamic visual acuity (Westheimer and McKee, 1975).

### Insights into the coordination between pursuit and saccades

Human and other primates follow moving objects with their eyes primarily using pursuit (Keller and Heinen, 1991; Krauzlis, 2004; Thier and Ilg, 2005; Lisberger, 2015; Kowler et al., 2019) but also leverage the evolutionarily older saccadic system as a supplement to compensate for shortcomings in the pursuit response (de Brouwer et al., 2002a). Shared target selection and coordination of pursuit and catch-up saccades (Orban de Xivry and Lefèvre, 2007) are enabled via the rostral Superior Colliculus (Basso et al., 2000; Krauzlis et al., 2000) and its feedback loops with downstream brainstem oculomotor output structures (Lee et al., 1988; for a review, see Sparks et al., 2002; van Opstal and Goossens, 2008; Takahashi et al., 2022) as well as its upward projections to cortical structures via the Pulvinar to potentially influence smooth corrections to pursuit (Berman and Wurtz, 2010).

Robinson (1975) first proposed a ballistic model based on precomputed parameters, which was subsequently extended by his laboratory and others (for a review, see Hafed, 2011) to include a feedback loop to monitor the ongoing saccade and thus to control the accuracy of the saccadic endpoint without the need for visual feedback, which cannot arrive on time given visual processing delays and the speed of saccades. More recently, Optican and colleagues have emphasized that multiple feedback loops through the brainstem and cerebellum control saccadic accuracy (Quaia et al., 1999; Pretegiani and Optican, 2017). Lastly, Lefevre and colleagues developed mechanistic model(s) specifically designed to capture the detailed visual signal processing that underlies the coordination between pursuit and catch-up saccades during steady-state tracking (de Brouwer et al., 2002a,b; Daye et al., 2014; Nachmani et al., 2020; Coutinho et al., 2021). Although our study does not address the computational details of any of these models, their shared essential structure (Hafed, 2011) nonetheless provides a helpful guide for identifying the key components of the neural computation responsible for this coordination.

At the highest level, the saccadic system generally needs to first decide which target to select from among many options in a real-world image (Krauzlis et al., 1999, 2004; Adler et al., 2002; Liston and Krauzlis, 2003; Case and Ferrera, 2007). In our paradigm, this is not an issue as there is only a single target and it has already been selected by the time steady-state tracking is engaged.

The next tier is the decision mechanism that triggers a catch-up saccade. This has been proposed to occur when the estimated future position error reaches a threshold (de Brouwer et al., 2002a,b; Daye et al., 2014; Nachmani et al., 2020; Coutinho et al., 2021). Our saccade-rate metric may shed some light on the status of that threshold. While the elevated rate observed in the ASL and EtOH conditions could simply reflect the increased demand caused by the overt pursuit deficit, the significant rate increases observed in the ASL+C and CSR conditions cannot, as they are not associated with impactful pursuit deficits. The rate increases observed in the two latter conditions therefore do not reflect the normal functioning of the saccadic system. While this could reflect non-adenosine-mediated components of fatigue/circadian responses in both these cases, the ASL+C effect could also be due to a direct non-specific effect of caffeine. While direct effects of caffeine have been observed on saccadic velocity (Connell et al., 2017), these effects appear quite small and become insignificant for small saccades (see their Figure 4D). Thus, caffeine may contribute to the observed time-awake-independent increase in ground recouped primarily via a large increase in saccade rate (Figure 5). Although there is no significant time-awake-dependent slope with caffeine, we did observe a significant increase in mean ground recouped with respect to baseline, see Figure 4B, as well as several hints of overcompensation, see Figures 3, 5, 6B. Thus, a parsimonious explanation is that all three manipulations of sleep (ASL, ASL+C, and CSR) cause a decrease in the threshold setting for catch-up saccade initiation, resulting in an increase in saccade rate regardless of changes in demand. This rate increase is compensatory during ASL, but not in the two other conditions, and is likely directly exacerbated by caffeine although, surprisingly, caffeine has been shown to decrease the rate of microsaccades (Hampsey et al., 2019). Future research is needed to tease apart the multifaceted effects of caffeine and other adenosine antagonists on eye movements.

The final tier is the saccade generation output pathway. Saccade amplitude, controlled by a sub-cortical motor execution system, shows the largest variation across conditions (Figure 5). EtOH shows a systematic increase in saccade amplitude which, along with the increase in rate, effects a nearly complete compensation for the lost ground due to the pursuit impairment. Thus, there is no indication that the catch-up saccade system is at all functionally impaired at the BACs below 0.07% tested, although some changes in the main-sequence dynamics have been reported (Roche and King, 2010; Schmitt et al., 2013; Tyson et al., 2021). ASL however, shows no systematic change in amplitude, despite the ongoing demand, resulting in incomplete compensation. This finding indicates a direct impairment in the control of saccade amplitude caused by sleep loss and/or circadian misalignment above and beyond the pursuit deficit. For ASL+C the residual pursuit deficit is of borderline significance, so the correction demand is minimal and inadequate to clearly determine if there is any residual saccadic impairment, but our circadian modulation analysis suggests that the saccadic system is highly effective under this condition. CSR also shows a pathological increase in rate in the absence of demand as pursuit is largely unimpaired. CSR, however, is also associated with a counterintuitive *decrease* in saccadic amplitude that is either a healthy compensatory response to the unneeded increase in saccade rate or a secondary pathology in saccadic generation. Thus, a parsimonious explanation is that ASL causes hypometric saccades that may (or may not) be present in the ASL+C and CSR conditions as well. To examine this issue more carefully, one would need to test a larger population or examine larger saccades than those occurring during our steady-state pursuit paradigm (e.g., by using higher velocity target motion).

### Possible neural underpinnings of the disparate effects of our stressors

A visual pathway from retina through V1 and to the Middle Temporal (MT) Area sends visual motion signals to pursuit pathways via the Medial Superior Temporal (MST) area (Dürsteler et al., 1987; Dürsteler and Wurtz, 1988; Newsome et al., 1988; Ilg and Thier, 2003; Ilg, 2008) and frontal (FPA) cortex (Tanaka and Lisberger, 2001; Chou and Lisberger, 2004) to sustain steady-state pursuit. The early motion pathway to MT also contributes to the computation of estimated future position error signal (Newsome et al., 1985), which upon reaching a threshold (de Brouwer et al., 2002a,b), triggers a saccadic motor response and drives the corrective saccadic accuracy. Our findings shed light on this coordination by illustrating an array of qualitatively different behavioral responses to different stressors that likely impact different neural structures that serve these various visuomotor sub-functions.

There are at least three components to the impairment and compensation processes observed in the current study:

First, all three stressors tested (EtOH, ASL, CSR) result in a significant reduction in steady-state pursuit gain, likely due to depressed responses somewhere within the regions of extrastriate and/or frontal cortex known to be responsible for driving steady-state pursuit. In particular, lesions in the interconnected Medial Superior Temporal (MST) area (Dürsteler et al., 1987) and Frontal Pursuit Area (FPA) of the Frontal Eye Fields (Morrow and Sharpe, 1995; Shi et al., 1998) have been shown to cause *sustained* uncorrected deficits in steady-state smooth eye speed, quite similar to those observed in this study (Compare our Figure 1 and Figure 5B of Tyson et al., 2021 with Figure 1B of Dürsteler et al., 1987 and Figure 1B of Shi et al., 1998). However, the reductions in pursuit gain in CSR and ASL+C are much smaller than that in our ASL or EtOH conditions, suggesting that adaptative processes (after chronic sleep loss) and inhibition of adenosine pathways (after caffeine administration) can ratchet pursuit gain back up, perhaps via the gain modulation identified in the FPA (Tanaka and Lisberger, 2001) or downstream from it (Chou and Lisberger, 2004), so as to counteract the effects of chronic or acute sleep disruption.

Second, all three stressors cause an increase in saccadic rate, which could be part of a direct compensatory response to weakened pursuit. However, in the cases of CSR and ASL+C, where compensation is largely unnecessary, the dramatic increase in rate is more likely due to the non-specific disruption of brainstem suppression of saccades by omnipause neurons (Rucker et al., 2011; Shinoda et al., 2011) as the observed rate increase is not necessary, and likely does not improve tracking.

Third, the largest divergence between the stressors occurs in the amplitude of compensatory saccades (Figure 5). In the EtOH condition, the saccadic system fully compensates by increasing the amplitude of catch-up saccades, so the most parsimonious explanation is that the midbrain and brainstem are unaffected by low-dose alcohol and the saccades themselves are healthy. During ASL, this is not the case as the small increase in saccade amplitude (that does not even reach significance) provides inadequate compensation. This is consistent with the systematic decrease in mean peak saccadic velocity observed under the ASL (De Gennaro et al., 2000; Stone et al., 2019), but not the EtOH (Tyson et al., 2021) condition. Thus, while there is a compensatory increase in the number of saccades in both conditions, saccades under the EtOH condition are properly implemented by the healthy performance of the rSC, burst neurons, and/or other components of the saccade generation output motor pathways, while those under the ASL condition fall short. This could be explained by two different mechanisms: (1) the inadequate neural integration of a reduced saccadic burst signal, consistent with the previous observations of a reduction in saccadic peak velocity although most models would predict slower yet accurate saccades if this were the case, or (2) impairment within the rSC, brainstem, and perhaps cerebellar feedback loops during execution of the catch-up saccade.

In sum, while our behavioral studies do not directly identify neural substrates, a simple straightforward explanation of our constellation of findings is that: (1) all four tested conditions exhibit impairment of extrastriate and/or frontal cortical visual motion responses driving pursuit, with CSR showing significant recuperative adaptation, perhaps via frontal cortical gain plasticity, and (2) the sleep and circadian altering conditions (even in the presence of caffeine) produce a decrease in omnipause-neuron output, thus non-specifically decreasing saccadic thresholds and maladaptively increasing saccade rate, and disrupt the rSC and/or its brainstem output pathways, thus impacting the control of saccadic amplitude. This latter effect may play a lesser role (if any) in CSR, again perhaps due to a recuperative adaptive process.

### Caffeine as a partial countermeasure

Our findings confirm that caffeine greatly ameliorates the time-awake-dependent effects of acute sleep loss. More specifically, caffeine (at 21.3 ± 4.8 mg/h) is an effective countermeasure for the time-awake sensitivity of ASL on pursuit with the observed sleep-dose-dependent effect indicating that, for this effect, caffeine is acting through adenosine-mediated neural pathways controlling homeostatic sleep drive. However, the absence of a significant sensitivity to time awake of the ground recouped yet an observed increase in the overall ground recouped across all nighttime hours, suggests a non-specific effect of caffeine directly on saccades. While there is prior evidence for such direct effects in unfatigued participants (Connell et al., 2017), these direct effects are limited (a small increase in the peak velocity of larger saccades) and we are not aware of any prior observations of caffeine effects on small catch-up saccade rate *per se.* Studies of caffeine on catch-up saccades of well-rested subjects as well as on the effects on larger saccades under ASL conditions are needed to resolve this issue.

### Insights into the interaction between homeostatic and circadian factors

Our method does not allow us to definitively segregate homeostatic and circadian effects. While our 24-hr acute sleep loss studies generated both homeostatic and circadian effects that we can tentatively segregate, especially with a caffeine-mitigation study that preferentially targets homeostatic sleep drive (see however, Blanco-Centurion et al., 2006), only a forced-desynchrony study (Dijk and Czeisler, 1995), which we did not perform, is able to do that conclusively. Thus, the simulated responses shown in Figure 7, based on the model in Figure 2, represent merely a self-consistency test and validation that the apparent circadian phase during ASL is perturbed by homeostatic effects that can be largely removed computationally to predict the observed ASL+C results where homeostatic effects were greatly reduced pharmacologically.

## Limitations

Our study was based on a set of small sample sizes (9–16 subjects for a given study), which limit the extrapolation to larger populations. However, our cohort sizes were large enough to show large and highly statistically significant effects across the oculometric suite, spanning a wide array of conditions (Stone et al., 2019; Evans et al., 2021; Tyson et al., 2021). Furthermore, due to the limited sample size and statistical power, linear mixed-effects models were not considered in our analysis.

Due the length of time needed for sleep-restricted participants to reach asymptotic performance, our CSR study was limited to a single dose (5 h/night) so our dose-response analysis (Figure 4) could not include a CSR condition.

Our discussion is based on a simplified framework of a catch-up saccade model and current saccadic models do not take into account that position errors can also drive corrective smooth accelerations that then preclude saccades (Carl and Gellman, 1987; Morris and Lisberger, 1987), a phenomenon that our stimulus paradigm and analyses do not address. Thus, any changes in the division of labor between these two mechanisms for correcting small positional errors during steady-state tracking were not addressed in this study.

Lastly, the proposed neural underpinnings for our behavioral observations are consistent with prior studies in non-human primates, but there are many possible alternate explanations. Nonetheless, they remain reasonable, educated, and constructive speculation at this juncture, to provide a potential basis for the design of future neurophysiological studies and aid in the interpretation of future clinical findings.

## Data availability statement

The data supporting the conclusions of this article will be made available by the authors, without undue reservation.

## Ethics statement

The studies involving human participants were reviewed and approved by National Aeronautics and Space Administration (NASA) Ames Research Center’s Human Research Institutional Review Board (HRIRB). The patients/participants provided their written informed consent to participate in this study.

## Author contributions

TT performed the data analysis and created all of the plots. TT and LS wrote the initial draft of the manuscript. All authors provided substantial contributions to the conception and design of the work and contributed to the revision and review of the submitted manuscript.

## References

[B1] Adler S. A. Bala J. Krauzlis R. J. (2002). Primacy of spatial information in guiding target selection for pursuit and saccades. *J. Vis.* 2 627–644. 10.1167/2.9.5 12678634

[B2] Barnes G. R. Asselman P. T. (1991). The mechanism of prediction in human smooth pursuit eye movements. *J. Physiol.* 439 439–461. 10.1113/jphysiol.1991.sp018675 1895243 PMC1180117

[B3] Basso M. A. Krauzlis R. J. Wurtz R. H. (2000). Activation and inactivation of rostral superior colliculus neurons during smooth-pursuit eye movements in monkeys. *J. Neurophysiol.* 84 892–908. 10.1152/jn.2000.84.2.892 10938315

[B4] Beck A. T. Steer R. A. (1993). *Beck depression inventory.* San Antonio, TX: Psychological Corporation.

[B5] Berman R. A. Wurtz R. H. (2010). Functional identification of a pulvinar path from superior colliculus to cortical area MT. *J. Neurosci.* 30 6342–6354. 10.1523/JNEUROSCI.6176-09.2010 20445060 PMC2919315

[B6] Beutter B. R. Stone L. S. (1998). Human motion perception and smooth eye movements show similar directional biases for elongated apertures. *Vis. Res.* 38 1273–1286. 10.1016/s0042-6989(97)00276-9 9666995

[B7] Beutter B. R. Stone L. S. (2000). Motion coherence affects human perception and pursuit similarly. *Vis. Neurosci.* 17 139–153. 10750835 10.1017/s0952523800171147

[B8] Blanco-Centurion C. Xu M. Murillo-Rodriguez E. Gerashchenko D. Shiromani A. M. Salin-Pascual R. J. (2006). Adenosine and sleep homeostasis in the basal forebrain. *J. Neurosci.* 26 8092–8100. 10.1523/JNEUROSCI.2181-06.2006 16885223 PMC6673779

[B9] Boman D. K. Hotson J. R. (1992). Predictive smooth pursuit eye movements near abrupt changes in motion direction. *Vis. Res.* 32 675–689. 10.1016/0042-6989(92)90184-k 1413552

[B10] Butcher J. N. Dahlstrom W. G. Graham J. R. Tellegen A. Kaemmer B. (1989). *Manual for the restandardized Minnesota multiphasic personality inventory: MMPI-2. An administrative and interpretive guide.* Minneapolis, MN: University of Minnesota Press.

[B11] Buysse D. J. Reynolds C. F. Monk T. H. Berman S. R. Kupfer D. J. (1989). The Pittsburgh sleep quality index: A new instrument for psychiatric practice and research. *Psychiatry Res.* 28 193–213. 10.1016/0165-1781(89)90047-4 2748771

[B12] Carl J. R. Gellman R. S. (1987). Human smooth pursuit: Stimulus-dependent responses. *J. Neurophysiol.* 57 1446–1463. 10.1152/jn.1987.57.5.1446 3585475

[B13] Case G. R. Ferrera V. P. (2007). Coordination of smooth pursuit and saccade target selection in monkeys. *J. Neurophysiol.* 98 2206–2214. 10.1152/jn.00021.2007 17715189

[B14] Castet E. Masson G. S. (2000). Motion perception during saccadic eye movements. *Nat. Neurosci.* 3 177–183. 10.1038/72124 10649574

[B15] Chou I. H. Lisberger S. G. (2004). The role of the frontal pursuit area in learning in smooth pursuit eye movements. *J. Neurosci.* 24 4124–4133. 10.1523/JNEUROSCI.0172-04.2004 15115807 PMC2553807

[B16] Connell C. J. W. Thompson B. Turuwhenua J. Hess R. F. Gant N. (2017). Caffeine increases the velocity of rapid eye movements in unfatigued humans. *Psychopharmacology* 234 2311–2323. 10.1007/s00213-017-4638-1 28536868

[B17] Coutinho J. D. Lefevre P. Blohm G. (2021). Confidence in predicted position error explains saccadic decisions during pursuit. *J. Neurophysiol.* 125 748–767. 10.1152/jn.00492.2019 33356899

[B18] Daye P. M. Blohm G. Lefevre P. (2014). Catch-up saccades in head-unrestrained conditions reveal that saccade amplitude is corrected using an internal model of target movement. *J. Vis.* 14:12. 10.1167/14.1.12 24424378 PMC4523018

[B19] de Brouwer S. Yuksel D. Blohm G. Missal M. Lefèvre P. (2002b). What triggers catch-up saccades during visual tracking? *J. Neurophysiol.* 87 1646–1650. 10.1152/jn.00432.2001 11877535

[B20] de Brouwer S. Missal M. Barnes G. Lefèvre P. (2002a). Quantitative analysis of catch-up saccades during sustained pursuit. *J. Neurophysiol.* 87 1772–1780. 10.1152/jn.00621.2001 11929898

[B21] De Gennaro L. Ferrara M. Urbani L. Bertini M. (2000). Oculomotor impairment after 1 night of total sleep deprivation: A dissociation between measures of speed and accuracy. *Clin. Neurophysiol.* 111 1771–1778. 10.1016/s1388-2457(00)00393-x 11018491

[B22] Derogatis L. R. (1994). *Symptom checklist-90-R: Administration, scoring & procedure manual for the revised version of the SCL-90.* Minneapolis, MN: National Computer Systems.

[B23] Dijk D. J. Czeisler C. A. (1995). Contribution of the circadian pacemaker and the sleep homeostat to sleep propensity, sleep structure, electroencephalographic slow waves, and sleep spindle activity in humans. *J. Neurosci.* 15(5 Pt 1) 3526–3538. 10.1523/JNEUROSCI.15-05-03526.1995 7751928 PMC6578184

[B24] Duffy J. F. Dijk D. J. (2002). Getting through to circadian oscillators: Why use constant routines? *J. Biol. Rhythms* 17 4–13. 10.1177/074873002129002294 11837947

[B25] Dürsteler M. R. Wurtz R. H. (1988). Pursuit and optokinetic deficits following chemical lesions of cortical areas MT and MST. *J. Neurophysiol.* 60 940–965. 10.1152/jn.1988.60.3.940 3171667

[B26] Dürsteler M. R. Wurtz R. H. Newsome W. T. (1987). Directional pursuit deficits following lesions of the foveal representation within the superior temporal sulcus of the macaque monkey. *J. Neurophysiol.* 57 1262–1287. 10.1152/jn.1987.57.5.1262 3585468

[B27] Evans E. Tyson T. Costedoat G. Stone L. (2021). The effects of chronic sleep restriction on human oculomotor behavior. *Sleep* 44(Suppl. 2):A54. 10.1093/sleep/zsab072.132

[B28] Fransson P. A. Modig F. Patel M. Gomez S. Magnusson M. (2010). Oculomotor deficits caused by 0.06% and 0.10% blood alcohol concentrations and relationship to subjective perception of drunkenness. *Clin. Neurophysiol.* 121 2134–2142. 10.1016/j.clinph.2010.05.003 20570556

[B29] Fransson P. A. Patel M. Magnusson M. Berg S. Almbladh P. Gomez S. (2008). Effects of 24-hour and 36-hour sleep deprivation on smooth pursuit and saccadic eye movements. *J. Vestib. Res.* 18 209–222. 19208965

[B30] Glickstein M. Gerrits N. Kralj-Hans I. Mercier B. Stein J. Voogd J. (1994). Visual pontocerebellar projections in the macaque. *J. Comp. Neurol.* 349 51–72. 10.1002/cne.903490105 7852626

[B31] Hafed Z. M. (2011). Mechanisms for generating and compensating for the smallest possible saccades. *Eur. J. Neurosci.* 33 2101–2113. 10.1111/j.1460-9568.2011.07694.x 21645104

[B32] Hafed Z. M. Krauzlis R. J. (2008). Goal representations dominate superior colliculus activity during extrafoveal tracking. *J. Neurosci.* 28 9426–9439. 10.1523/JNEUROSCI.1313-08.2008 18799675 PMC2698013

[B33] Hafed Z. M. Goffart L. Krauzlis R. J. (2009). A neural mechanism for microsaccade generation in the primate superior colliculus. *Science* 323 940–943. 10.1126/science.1166112 19213919 PMC2655118

[B34] Hampsey E. Overton P. G. Stafford T. (2019). Microsaccade rate as a measure of drug response. *J. Eye Mov. Res.* 12 1–10. 10.16910/jemr.12.6.12 33828750 PMC7962677

[B35] Heinen S. J. Badler J. B. Watamaniuk S. N. J. (2018). Choosing a foveal goal recruits the saccadic system during smooth pursuit. *J. Neurophysiol.* 120 489–496. 10.1152/jn.00418.2017 29668381 PMC6139446

[B36] Heinen S. J. Potapchuk E. Watamaniuk S. N. (2016). A foveal target increases catch-up saccade frequency during smooth pursuit. *J. Neurophysiol.* 115 1220–1227. 10.1152/jn.00774.2015 26631148 PMC4808105

[B37] Horne J. A. Ostberg O. (1976). A self-assessment questionnaire to determine morningness-eveningness in human circadian rhythms. *Int. J. Chronobiol.* 4 97–110. 1027738

[B38] Ilg U. J. (2008). The role of areas MT and MST in coding of visual motion underlying the execution of smooth pursuit. *Vis. Res.* 48 2062–2069. 10.1016/j.visres.2008.04.015 18508104

[B39] Ilg U. J. Thier P. (2003). Visual tracking neurons in primate area MST are activated by smooth-pursuit eye movements of an “imaginary” target. *J. Neurophysiol.* 90 1489–1502. 10.1152/jn.00272.2003 12736240

[B40] Keller E. L. Heinen S. J. (1991). Generation of smooth-pursuit eye movements: Neuronal mechanisms and pathways. *Neurosci. Res.* 11 79–107. 10.1016/0168-0102(91)90048-4 1656345

[B41] Keller E. L. Missal M. (2003). Shared brainstem pathways for saccades and smooth-pursuit eye movements. *Ann. N. Y. Acad. Sci.* 1004 29–39. 10.1196/annals.1303.004 14662445

[B42] Kheradmand A. Zee D. S. (2011). Cerebellum and ocular motor control. *Front. Neurol.* 2:53. 10.3389/fneur.2011.00053 21909334 PMC3164106

[B43] Kowler E. Rubinstein J. F. Santos E. M. Wang J. (2019). Predictive smooth pursuit eye movements. *Annu. Rev. Vis. Sci.* 5 223–246. 10.1146/annurev-vision-091718-014901 31283450

[B44] Krauzlis R. J. (2004). Recasting the smooth pursuit eye movement system. *J. Neurophysiol.* 91 591–603. 10.1152/jn.00801.2003 14762145

[B45] Krauzlis R. J. Adler S. A. (2001). Effects of directional expectations on motion perception and pursuit eye movements. *Vis. Neurosci.* 18 365–376. 11497413 10.1017/s0952523801183033

[B46] Krauzlis R. J. Lisberger S. G. (1991). Visual motion commands for pursuit eye movements in the cerebellum. *Science* 253 568–571. 10.1126/science.1907026 1907026

[B47] Krauzlis R. J. Miles F. A. (1998). Role of the oculomotor vermis in generating pursuit and saccades: Effects of microstimulation. *J. Neurophysiol.* 80 2046–2062. 10.1152/jn.1998.80.4.2046 9772260

[B48] Krauzlis R. J. Stone L. S. (1999). Tracking with the mind’s eye. *Trends Neurosci.* 22 544–550. 10.1016/s0166-2236(99)01464-2 10542434

[B49] Krauzlis R. J. Basso M. A. Wurtz R. H. (2000). Discharge properties of neurons in the rostral superior colliculus of the monkey during smooth-pursuit eye movements. *J. Neurophysiol.* 84 876–891. 10.1152/jn.2000.84.2.876 10938314

[B50] Krauzlis R. J. Liston D. Carello C. D. (2004). Target selection and the superior colliculus: Goals, choices and hypotheses. *Vis. Res.* 44 1445–1451. 10.1016/j.visres.2004.01.005 15066403

[B51] Krauzlis R. J. Zivotofsky A. Z. Miles F. A. (1999). Target selection for pursuit and saccadic eye movements in humans. *J. Cogn. Neurosci.* 11 641–649. 10.1162/089892999563706 10601745

[B52] Krukowski A. E. Stone L. S. (2005). Expansion of direction space around the cardinal axes revealed by smooth pursuit eye movements. *Neuron* 45 315–323. 10.1016/j.neuron.2005.01.005 15664182

[B53] Krukowski A. E. Begault D. R. Wenzel E. M. Stone L. S. (2002). “Human smooth pursuit eye movement responses to visual, auditory, and imagined target motion,” in *Proceedings of the 12th annual meeting, the society for the neural control of movement*, Naples. 10.1152/jn.00611.2003

[B54] Krukowski A. E. Pirog K. A. Beutter B. R. Brooks K. R. Stone L. S. (2003). Human discrimination of visual direction of motion with and without smooth pursuit eye movements. *J. Vis.* 3 831–840. 10.1167/3.11.16 14765965

[B55] Lee C. Rohrer W. H. Sparks D. L. (1988). Population coding of saccadic eye movements by neurons in the superior colliculus. *Nature* 332 357–360. 10.1038/332357a0 3352733

[B56] Lehtinen I. Lang A. H. Jantti V. Keskinen E. (1979). Acute effects of alcohol on saccadic eye movements. *Psychopharmacology* 63 17–23. 112616 10.1007/BF00426915

[B57] Leigh J. R. Zee D. S. (2006). *The neurology of eye movements.* Oxford: Oxford University Press.

[B58] Lisberger S. G. (2015). Visual guidance of smooth pursuit eye movements. *Annu. Rev. Vis. Sci.* 1 447–468. 10.1146/annurev-vision-082114-035349 28532366

[B59] Lisberger S. G. Westbrook L. E. (1985). Properties of visual inputs that initiate horizontal smooth pursuit eye movements in monkeys. *J. Neurosci.* 5 1662–1673. 4009252 10.1523/JNEUROSCI.05-06-01662.1985 PMC6565252

[B60] Liston D. B. Stone L. S. (2014). Oculometric assessment of dynamic visual processing. *J. Vis.* 14:12. 10.1167/14.14.12 25527150

[B61] Liston D. B. Krukowski A. E. Stone L. S. (2013). Saccade detection during smooth tracking. *Displays* 34 171–176.

[B62] Liston D. B. Simpson S. Wong L. R. Rich M. Stone L. S. (2016). “Design and validation of a simple eye-tracking system,” in *Proceedings of the 9th biennial ACM symposium on eye tracking research & applications*, New York, NY, 221–224.

[B63] Liston D. B. Wong L. R. Stone L. S. (2017). Oculometric assessment of sensorimotor impairment associated with TBI. *Optom. Vis. Sci.* 94 51–59. 10.1097/OPX.0000000000000918 27391532 PMC5428838

[B64] Liston D. Krauzlis R. J. (2003). Shared response preparation for pursuit and saccadic eye movements. *J. Neurosci.* 23 11305–11314. 14672994 10.1523/JNEUROSCI.23-36-11305.2003 PMC6740528

[B65] MacAvoy M. G. Gottlieb J. P. Bruce C. J. (1991). Smooth-pursuit eye movement representation in the primate frontal eye field. *Cereb. Cortex* 1 95–102. 10.1093/cercor/1.1.95 1822728

[B66] May J. G. Keller E. L. Suzuki D. A. (1988). Smooth-pursuit eye movement deficits with chemical lesions in the dorsolateral pontine nucleus of the monkey. *J. Neurophysiol.* 59 952–977. 10.1152/jn.1988.59.3.952 3367205

[B67] Miles F. A. Fuller J. H. (1975). Visual tracking and the primate flocculus. *Science* 189 1000–1002. 10.1126/science.1083068 1083068

[B68] Morris E. J. Lisberger S. G. (1987). Different responses to small visual errors during initiation and maintenance of smooth-pursuit eye movements in monkeys. *J. Neurophysiol.* 58 1351–1369. 10.1152/jn.1987.58.6.1351 3437336

[B69] Morrow M. J. Sharpe J. A. (1995). Deficits of smooth-pursuit eye movement after unilateral frontal lobe lesions. *Ann. Neurol.* 37 443–451. 10.1002/ana.410370406 7717680

[B70] Moschovakis A. K. Kitama T. Dalezios Y. Petit J. Brandi A. M. Grantyn A. A. (1998). An anatomical substrate for the spatiotemporal transformation. *J. Neurosci.* 18 10219–10229. 9822775 10.1523/JNEUROSCI.18-23-10219.1998 PMC6793294

[B71] Movshon J. A. Newsome W. T. (1996). Visual response properties of striate cortical neurons projecting to area MT in macaque monkeys. *J. Neurosci.* 16 7733–7741. 8922429 10.1523/JNEUROSCI.16-23-07733.1996 PMC6579106

[B72] Munoz D. P. Wurtz R. H. (1993a). Fixation cells in monkey superior colliculus. I. Characteristics of cell discharge. *J. Neurophysiol.* 70 559–575. 10.1152/jn.1993.70.2.559 8410157

[B73] Munoz D. P. Wurtz R. H. (1993b). Fixation cells in monkey superior colliculus. II. Reversible activation and deactivation. *J. Neurophysiol.* 70 576–589. 10.1152/jn.1993.70.2.576 8410158

[B74] Mustari M. J. Fuchs A. F. Wallman J. (1988). Response properties of dorsolateral pontine units during smooth pursuit in the rhesus macaque. *J. Neurophysiol.* 60 664–686. 10.1152/jn.1988.60.2.664 3171646

[B75] Nachmani O. Coutinho J. Khan A. Z. Lefevre P. Blohm G. (2020). Predicted position error triggers catch-up saccades during sustained smooth pursuit. *eNeuro* 7 1–22. 10.1523/ENEURO.0196-18.2019 31862791 PMC6964921

[B76] Newsome W. T. Paré E. B. (1988). A selective impairment of motion perception following lesions of the middle temporal visual area (MT). *J. Neurosci.* 8 2201–2211. 3385495 10.1523/JNEUROSCI.08-06-02201.1988 PMC6569328

[B77] Newsome W. T. Wurtz R. H. Dürsteler M. R. Mikami A. (1985). Deficits in visual motion processing following ibotenic acid lesions of the middle temporal visual area of the macaque monkey. *J. Neurosci.* 5 825–840. 3973698 10.1523/JNEUROSCI.05-03-00825.1985 PMC6565029

[B78] Newsome W. T. Wurtz R. H. Komatsu H. (1988). Relation of cortical areas MT and MST to pursuit eye movements. II. Differentiation of retinal from extraretinal inputs. *J. Neurophysiol*. 60, 604–620. 10.1152/jn.1988.60.2.604 3171644

[B79] Optican L. M. Pretegiani E. (2017). What stops a saccade? *Philos. Trans. R. Soc. Lond. B Biol. Sci.* 372:20160194. 10.1098/rstb.2016.0194 28242728 PMC5332853

[B80] Orban de Xivry J. J. Lefèvre P. (2007). Saccades and pursuit: Two outcomes of a single sensorimotor process. *J. Physiol.* 584(Pt 1) 11–23. 10.1113/jphysiol.2007.139881 17690138 PMC2277072

[B81] Pidcoe P. E. Wetzel P. A. (2006). Oculomotor tracking strategy in normal subjects with and without simulated scotoma. *Invest. Ophthalmol. Vis. Sci.* 47 169–178. 10.1167/iovs.04-0564 16384959

[B82] Pretegiani E. Optican L. M. (2017). Eye movements in Parkinson’s disease and inherited Parkinsonian syndromes. *Front. Neurol.* 8:592. 10.3389/fneur.2017.00592 29170650 PMC5684125

[B83] Quaia C. Lefevre P. Optican L. M. (1999). Model of the control of saccades by superior colliculus and cerebellum. *J. Neurophysiol.* 82 999–1018. 10.1152/jn.1999.82.2.999 10444693

[B84] Ramat S. Leigh R. J. Zee D. S. Optican L. M. (2007). What clinical disorders tell us about the neural control of saccadic eye movements. *Brain* 130(Pt 1) 10–35. 10.1093/brain/awl309 17121745

[B85] Rashbass C. (1961). The relationship between saccadic and smooth tracking eye movements. *J. Physiol.* 159 326–338. 14490422 10.1113/jphysiol.1961.sp006811 PMC1359508

[B86] Robinson D. A. (1975). “Oculomotor control signals,” in *Basic mechanisms of ocular motility and their clinical implications*, eds Lennerstrand G. Bach-y-Rita P. (Oxford: Pergamon Press), 337–374.

[B87] Roche D. J. King A. C. (2010). Alcohol impairment of saccadic and smooth pursuit eye movements: Impact of risk factors for alcohol dependence. *Psychopharmacology* 212 33–44. 10.1007/s00213-010-1906-8 20635179 PMC4633411

[B88] Rucker J. C. Ying S. H. Moore W. Optican L. M. Buttner-Ennever J. Keller E. L. (2011). Do brainstem omnipause neurons terminate saccades? *Ann. N. Y. Acad. Sci.* 1233 48–57. 10.1111/j.1749-6632.2011.06170.x 21950975 PMC3438674

[B89] Schmitt K. U. Lanz C. Muser M. H. Walz F. Schwarz U. (2013). Saccadic eye movements after low-dose oral alcohol exposure. *J. Forensic Leg Med.* 20 870–874. 10.1016/j.jflm.2013.06.023 24112339

[B90] Shanidze N. Ghahghaei S. Verghese P. (2016). Accuracy of eye position for saccades and smooth pursuit. *J. Vis.* 16:23. 10.1167/16.15.23 28006073 PMC5213993

[B91] Shi D. Friedman H. R. Bruce C. J. (1998). Deficits in smooth-pursuit eye movements after muscimol inactivation within the primate’s frontal eye field. *J. Neurophysiol.* 80 458–464. 10.1152/jn.1998.80.1.458 9658064

[B92] Shinoda Y. Sugiuchi Y. Takahashi M. Izawa Y. (2011). Neural substrate for suppression of omnipause neurons at the onset of saccades. *Ann. N. Y. Acad. Sci.* 1233 100–106. 10.1111/j.1749-6632.2011.06171.x 21950982

[B93] Sparks D. L. Barton E. J. Gandhi N. J. Nelson J. (2002). Studies of the role of the paramedian pontine reticular formation in the control of head-restrained and head-unrestrained gaze shifts. *Ann. N. Y. Acad. Sci.* 956 85–98. 10.1111/j.1749-6632.2002.tb02811.x 11960796

[B94] Spielberger C. D. Gorsuch R. L. Lushene R. Vagg P. R. Jacobs G. A. (1983). *Manual for the state-trait anxiety inventory.* Palo Alto, CA: Consulting Psychologists Press.

[B95] Steinbach M. J. (1976). Pursuing the perceptual rather than the retinal stimulus. *Vis. Res.* 16 1371–1376. 10.1016/0042-6989(76)90154-1 1007015

[B96] Stone L. S. Krauzlis R. J. (2003). Shared motion signals for human perceptual decisions and oculomotor actions. *J. Vis.* 3 725–736. 10.1167/3.11.7 14765956

[B97] Stone L. S. Lisberger S. G. (1990a). Visual responses of Purkinje cells in the cerebellar flocculus during smooth-pursuit eye movements in monkeys. I. Simple spikes. *J. Neurophysiol.* 63 1241–1261. 10.1152/jn.1990.63.5.1241 2358872

[B98] Stone L. S. Lisberger S. G. (1990b). Visual responses of Purkinje cells in the cerebellar flocculus during smooth-pursuit eye movements in monkeys. II. Complex spikes. *J. Neurophysiol.* 63 1262–1275. 10.1152/jn.1990.63.5.1262 2358873

[B99] Stone L. S. Beutter B. R. Lorenceau J. (2000). Visual motion integration for perception and pursuit. *Perception* 29 771–787. 10.1068/p2979 11064800

[B100] Stone L. S. Tyson T. L. Cravalho P. F. Feick N. H. Flynn-Evans E. E. (2019). Distinct pattern of oculomotor impairment associated with acute sleep loss and circadian misalignment. *J. Physiol.* 597 4643–4660. 10.1113/JP277779 31389043 PMC6852126

[B101] Suzuki D. A. Keller E. L. (1984). Visual signals in the dorsolateral pontine nucleus of the alert monkey: Their relationship to smooth-pursuit eye movements. *Exp. Brain Res.* 53 473–478. 10.1007/BF00238178 6705876

[B102] Suzuki D. A. Keller E. L. (1988a). The role of the posterior vermis of monkey cerebellum in smooth-pursuit eye movement control. I. Eye and head movement-related activity. *J. Neurophysiol.* 59 1–18. 10.1152/jn.1988.59.1.1 3343598

[B103] Suzuki D. A. Keller E. L. (1988b). The role of the posterior vermis of monkey cerebellum in smooth-pursuit eye movement control. II. Target velocity-related Purkinje cell activity. *J. Neurophysiol.* 59 19–40. 10.1152/jn.1988.59.1.19 3343601

[B104] Takagi M. Zee D. S. Tamargo R. J. (2000). Effects of lesions of the oculomotor cerebellar vermis on eye movements in primate: Smooth pursuit. *J. Neurophysiol.* 83 2047–2062. 10.1152/jn.2000.83.4.2047 10758115

[B105] Takahashi M. Sugiuchi Y. Na J. Shinoda Y. (2022). Brainstem circuits triggering saccades and fixation. *J. Neurosci.* 42 789–803. 10.1523/JNEUROSCI.1731-21.2021 34880121 PMC8808722

[B106] Tanaka M. Lisberger S. G. (2001). Regulation of the gain of visually guided smooth-pursuit eye movements by frontal cortex. *Nature* 409 191–194. 10.1038/35051582 11196642

[B107] Thier P. Ilg U. J. (2005). The neural basis of smooth-pursuit eye movements. *Curr. Opin. Neurobiol.* 15 645–652. 10.1016/j.conb.2005.10.013 16271460

[B108] Tychsen L. Lisberger S. G. (1986). Visual motion processing for the initiation of smooth-pursuit eye movements in humans. *J. Neurophysiol.* 56 953–968. 10.1152/jn.1986.56.4.953 3783238

[B109] Tyson T. L. Feick N. H. Cravalho P. F. Flynn-Evans E. E. Stone L. S. (2019). Differential effects of low-dose alcohol versus acute sleep deprivation on light-evoked pupil response dynamics. *J. Vis.* 19:73a. 10.1167/19.10.73a

[B110] Tyson T. L. Feick N. H. Cravalho P. F. Flynn-Evans E. E. Stone L. S. (2021). Dose-dependent sensorimotor impairment in human ocular tracking after acute low-dose alcohol administration. *J. Physiol.* 599 1225–1242. 10.1113/JP280395 33332605 PMC7898833

[B111] van Opstal A. J. Goossens H. H. (2008). Linear ensemble-coding in midbrain superior colliculus specifies the saccade kinematics. *Biol. Cybern.* 98 561–577. 10.1007/s00422-008-0219-z 18491166 PMC2798131

[B112] Vorstius C. Radach R. Lang A. R. Riccardi C. J. (2008). Specific visuomotor deficits due to alcohol intoxication: Evidence from the pro- and antisaccade paradigms. *Psychopharmacology* 196 201–210. 10.1007/s00213-007-0954-1 17982744

[B113] Westheimer G. McKee S. P. (1975). Visual acuity in the presence of retinal-image motion. *J. Opt. Soc. Am.* 65 847–850. 10.1364/josa.65.000847 1142031

[B114] Wurtz R. H. Optican L. M. (1994). Superior colliculus cell types and models of saccade generation. *Curr. Opin. Neurobiol.* 4 857–861. 10.1016/0959-4388(94)90134-1 7888769

[B115] Wyatt J. K. Cajochen C. Ritz-De Cecco A. Czeisler C. A. Dijk D. J. (2004). Low-dose repeated caffeine administration for circadian-phase-dependent performance degradation during extended wakefulness. *Sleep* 27 374–381. 10.1093/sleep/27.3.374 15164887

[B116] Zee D. S. Yamazaki A. Butler P. H. Gucer G. (1981). Effects of ablation of flocculus and paraflocculus on eye movements in primate. *J. Neurophysiol.* 46 878–899. 10.1152/jn.1981.46.4.878 7288469

[B117] Zils E. Sprenger A. Heide W. Born J. Gais S. (2005). Differential effects of sleep deprivation on saccadic eye movements. *Sleep* 28 1109–1115. 16268380 10.1093/sleep/28.9.1109

